# CHALLENGING IDEAS OF CONSTANT CONNECTION AND DIGITAL INCLUSION

**DOI:** 10.1093/geroni/igad104.0820

**Published:** 2023-12-21

**Authors:** Shelia Cotten, Amy Schuster

**Affiliations:** Clemson University, Clemson, South Carolina, United States; Clemson University, Clemson, South Carolina, United States

## Abstract

1We live in a world where we are constantly connected to devices. This constant digital connection is where many individuals communicate and exchange social support with social ties. For most, this results in being online and connected to devices multiple times each day. Older adults have been slower to adopt and use emerging technologies. This presentation challenges the concept of digital inclusion by focusing on older adults and their technology use. We provide an overview of technology usage by different age groups using existing national level data. Next, we utilize life course and aging theoretical perspectives to help articulate that while a constant connection to devices may be normative for younger age groups, this may not, and perhaps should not, be the case for older adults. We conclude with a discussion of the social construction of digital inclusion and emphasize the significant variation that exists in this construct.

